# Particle Filters: A Hands-On Tutorial

**DOI:** 10.3390/s21020438

**Published:** 2021-01-09

**Authors:** Jos Elfring, Elena Torta, René van de Molengraft

**Affiliations:** 1 Department of Mechanical Engineering, Eindhoven University of Technology, 5612 AZ Eindhoven, The Netherlands; e.torta@tue.nl (E.T.); M.J.G.v.d.Molengraft@tue.nl (R.v.d.M.); 2 Product Unit Autonomous Driving, TomTom, 1011 AC Amsterdam, The Netherlands

**Keywords:** particle filter, auxiliary, adaptive, extended Kalman, tutorial

## Abstract

The particle filter was popularized in the early 1990s and has been used for solving estimation problems ever since. The standard algorithm can be understood and implemented with limited effort due to the widespread availability of tutorial material and code examples. Extensive research has advanced the standard particle filter algorithm to improve its performance and applicability in various ways in the years after. As a result, selecting and implementing an advanced version of the particle filter that goes beyond the standard algorithm and fits a specific estimation problem requires either a thorough understanding or reviewing large amounts of the literature. The latter can be heavily time consuming especially for those with limited hands-on experience. Lack of implementation details in theory-oriented papers complicates this task even further. The goal of this tutorial is facilitating the reader to familiarize themselves with the key concepts of advanced particle filter algorithms and to select and implement the right particle filter for the estimation problem at hand. It acts as a single entry point that provides a theoretical overview of the filter, its assumptions and solutions for various challenges encountered when applying particle filters. Besides that, it includes a running example that demonstrates and implements many of the challenges and solutions.

## 1. Introduction

This paper is a hands-on particle filter tutorial. Similar to the seminal work of [1], the focus of this tutorial is on applying a particle filter for estimation problems. Applications that require estimating quantities occur in many domains:

- Estimating the position and orientation of a vehicle with respect to a map using measurements of landmarks whose positions are known from that map [2].
- Estimating asset returns in econometrics [3].
- Building a map of a robot’s environment while navigating this environment [4].
- Fault detection by state estimation, e.g., in a chemical process [5].

In the context of particle filters, quantities that must be estimated are collected in a ‘state vector’. In the localization example above, the state vector contains the position and orientation of a vehicle with respect to a map, whereas the econometrics example involves a state vector that represents the value of an asset. Due to the dynamic nature of the state in these examples, estimates are computed recursively over time: whenever new information is available the estimates are updated based on this information. New information is delivered by one or more sensors that measure quantities related to the state vector. For that reason, this tutorial focuses on using a particle filter for recursively estimating dynamic states using measurements, as demonstrated earlier by [1].

Quickly getting up to speed with a particle filter and at the same time exploiting many of the advances made since the work of [1] requires a tremendous effort in terms of both reviewing relevant work and implementing the algorithms. Tutorials available in the scientific literature, such as [6,7], describe mathematical aspects much more thoroughly than practical challenges that appear when applying particle filters for real-world problems. At the same time, such tutorials favor a detailed description of some methods over a broad overview that mainly explains the details needed to solve challenges at hand. Public repositories [8,9] on the other hand often contain code examples that can easily be executed, which is advantageous for those interested in applying a particle filter to practical problems. However, often a single version of the algorithm is implemented and background information or a structured overview of possible shortcomings of methods and how to overcome them is missing. The goal of this tutorial is bridging this gap between theory and implementation.

This work is meant as a single entry-point that speeds up development in estimation problems for which particle filters are considered. It combines a broad overview of the scientific literature with code that implements both the standard particle filter and many of the successful advances proposed in the last decades. Five challenges relevant to anyone adopting a particle filter for a real-world problem are identified. Each of the challenges is explained and various options for solving it are presented. Theoretical and practical aspects of solutions are described together with references for further reading. In order to enable a better understanding of the concepts and challenges, a running example is used throughout the manuscript. The code related to the running example is available in a public repository [10] and reveals implementation details that may be difficult to grasp based on mathematical descriptions or pseudo code only. Readers are encouraged to download the code and run the algorithms. It allows for experimenting with different particle filter variants and settings.

## 2. Related Work

This section reviews tutorial and overview papers that have a focus on theoretical or practical aspects of particle filters. For textbooks with elaborate sections on particle filters the reader is referred to [11,12,13].

The first group of works are more extensive in multiple ways [6,7,14,15,16,17].

- Their focus is broader, e.g., nonlinear filtering in general, instead of just particle filters.
- Mathematical details and derivations are described in a more elaborate way.

This work only explains the mathematics needed to understand the concepts and focuses on particle filters exclusively. This way, room has been made for explaining aspects that are not included in the above mentioned works.

- A list of challenges encountered when applying particle filters and an extensive overview of advanced particle filters that can be used to address these challenges.
- A running example (and code), such that differences of advanced particle filters can be illustrated.

The work of [14] presents an elaborate textbook style overview of estimation algorithms. Its focus is more theory-oriented and it is a recommended read for people looking for a broad overview. It does not include a running example, and is less elaborate on advanced particle filters. It is much less elaborate in terms of particle filter specific code examples.

Both [6,7] are more extensive in terms of mathematical derivations and proofs. These works are recommended for readers looking for mathematical background information. Furthermore, a comparison between particle filters and other popular estimation algorithms, such as Kalman filters, is included in [6]. Practical challenges and advanced particle filter solutions to these problems are treated in a less elaborate manner and only pseudo-code is given.

The work of [15] spans both a broader range of algorithms and problems. Besides recursive estimation of dynamic states it includes, e.g., estimation of static states and smoothing. The work is less elaborate on different types of advanced particle filters and how these can be used to handle specific challenges and does not include code.

Finally, [16,17] are much more compact on particle filters, challenges and implementation and broader in terms of focus, i.e., (nonlinear) filtering in general.

The second group of papers applies particle filter theory to problems in a specific application domain. The particle filter specific part is compact compared to this work and more room is given to application related challenges and solutions [5,18,19,20,21,22,23]. The work of [18] focuses on the passive synthetic aperture problem in an uncertain ocean environment. The work of [19] demonstrates how particle filters can be used for resolving problems in wireless communication and [20] presents recent advances in the context of multitarget tracking. Mobile robot localization aspects and models are explained in [21], whereas [5] presents particle filters in the context of fault detection and diagnosis of hybrid systems. An elaborate tutorial on particle filters in the context of psychology can be found in [22]. Finally, [23] benchmarks particle filters settings for vehicle localization using data from lidar sensors.

The third group of works surveys one particular aspect of the particle filter [24,25,26,27,28,29,30]. This tutorial addresses each of these aspects, albeit less extensively. References to these works will be repeated in later sections explaining the aspect at hand. The work of [24] explains how graphical processing units can be used to speed up the computation of particle filters. Resampling algorithms (what is meant with resampling and why it is needed will be explained in Section 5.1) are classified and explained in [25], whereas [26,27] compare some of the most popular resampling schemes. The work of [30] reviews ways to fight sample degeneracy and impoverishment (the sample degeneracy and impoverishment problems will be explained in Section 4.2.1 and Section 4.2.2). In [28], it is explained how roughening methods can be used to prevent sample impoverishment. Methods for changing the number of particles on the fly are surveyed in [29].

In summary, this tutorial differs from the three groups of related works in different ways.

1. The focus is on particle filters only which allows for an in-depth description without focusing on one specific aspect of the filter.
2. Theory and implementation are described equally thorough.
3. The description is domain independent.

The source code accompanying the paper includes a simulation that will be used as running example throughout the entire manuscript. It is meant to ease further understanding of both theory and implementation. The running example is separated from the main text such that it can easily be skipped if preferred: whenever we switch from main text to running example the start and end are indicated by Example and □ respectively. References to code that can be used to reproduce the results will be given as well.

The paper continues with the problem statement in Section 3. Then, Section 4 introduces the particle filter and identifies five important challenges that should be considered when using particle filters for real world problems. Section 5 explains ways to solve the challenges and conclusions are drawn in Section 6.

## 3. Problem Statement

### 3.1. Conceptual Problem Statement

This tutorial assumes the reader wants to solve a recursive state estimation problem by using a particle filter. The goal is to estimate a state vector *x*. More specifically, the goal is to track the hidden state sequence $\left\{x_{k}\right\}$ of a dynamical system, where k∈N is a discrete time step and N is the set of natural numbers: 1,2,3,…. The word hidden emphasizes that no direct state measurements are available. For estimating the state, two sources of information are required.

1. A process model that encodes prior knowledge on how the state $x_{k}$ is expected to evolve over time.
2. A measurement model that relates measurements to the state $x_{k}$.

These two sources of information are formalized by mathematical models that can be nonlinear. The process model reflects how the state changes over time given noise and optional inputs:

(1) $$x_{k}=f_{k}\left(x_{k-1},u_{k},v_{k-1}\right),$$

where $f_{k}$ is a function that uniquely associates the state at time step k−1 with a state at time step *k*. The process model noise sequence $v_{k-1}$ is independent and identically distributed. It represents uncertainties related to the process model, e.g., as a result of model related simplifications. Depending on the application, a deterministic control input $u_{k}$ can be present.

The second source of information is the measurement model:

(2) $$z_{k}=h_{k}\left(x_{k},u_{k},n_{k}\right),$$

where $h_{k}$ is a function that associates the state with an expected measurement. Here $n_{k}$ is an independent and identically distributed noise sequence representing the measurement noise. In case multiple sensors are present, each sensor can be associated with its own measurement model.

In the remainder of this tutorial the Bayesian perspective is adopted for explaining the particle filter. The state vector is represented by a random variable and a probability distribution over that variable, sometimes referred to as belief, represents the uncertainty of the estimate. Bayes’ theorem will be used to refine the belief based on a prior estimate and newly received measurements. This tutorial is meant to be understandable without prior knowledge on Bayesian statistics. The interested reader is referred to [31] for a more elaborate introduction of Bayesian statistics or probability theory in general.

The popularity of the particle filter arises from the fact that, contrary to other state estimation techniques, such as Kalman filter [6], both linear and nonlinear process and measurement models can be used. In addition, the filter enables representing state estimates with arbitrarily shaped probability distributions as will be explained in Section 4.1.

**Figure 1:**
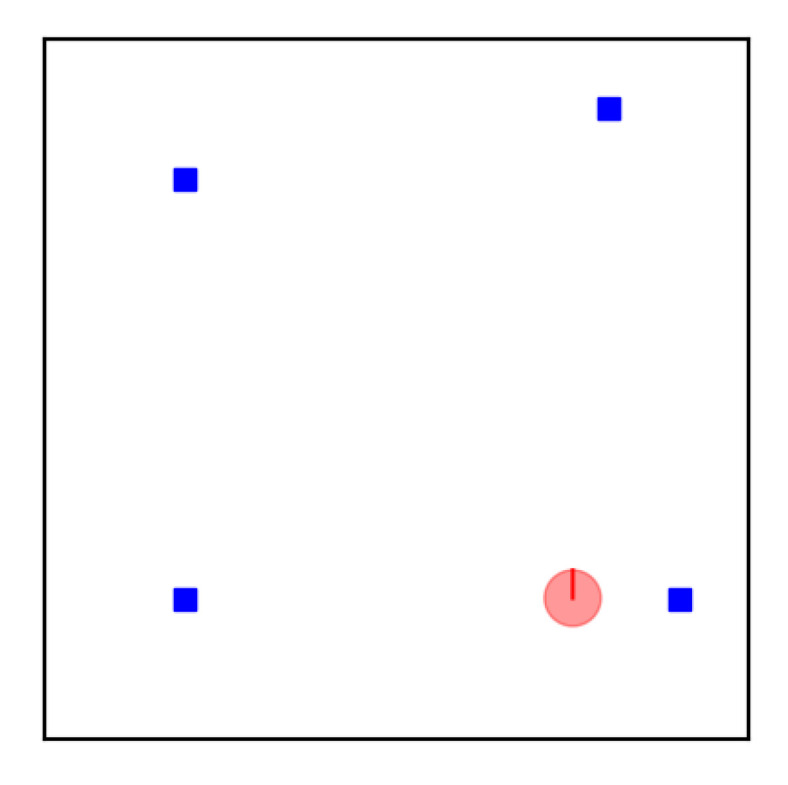
Simulation environment. The red circle represents the 2D robot position, the red line within the circle represents the robot’s heading, the blue rectangles represent the landmarks that are detected by the robot.

**Example** **1.** *The running example used throughout this manuscript is a robot localization problem. The robot moves around in a 2D world and uses a map to localize itself with respect to the map origin (lower left corner). The state vector x contains the 2D pose of the robot, i.e., its 2D position in an x–y coordinate frame (expressed in meters) and its orientation with respect to this frame (expressed in radians). The orientation will also be referred to as heading or heading angle. A figure showing the simulated robot (red sphere) in a simulated world containing four landmarks (blue rectangles) is shown in Figure 1.*

**Figure 2:**
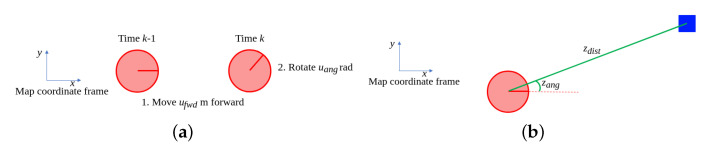
Visualizations of the process and measurement models used in the running example. The robot pose is denoted by the red circle, the landmarks are denoted by blue rectangles. (**a**) Process model used in the running example: the robot at time k−1 on the left, predicted robot pose at time *k* on the right. (**b**) Measurement model used in the running example. Measurement $z_{ang}$ is defined with respect to the *x*-axis of the map coordinate frame. As a result, measurements are independent of the robot’s heading.

*In the running example [10], the robot’s forward displacement $u_{fwd}$ is measured in meters and its change in rotation $u_{ang}$ is measured in radians. For formulating a process model, it is assumed that the robot first moves forward $u_{fwd}$ m in the direction of its heading and then rotates $u_{ang}$ rad, as is shown in Figure 2a. The resulting process model therefore is:*

(3) $$\underset{x_{k}}{\underset{︸}{{\left[\begin{array}{c} x_{r}\\ y_{r}\\ \theta_{r} \end{array}\right]}_{k}}}=\underset{x_{k-1}}{\underset{︸}{{\left[\begin{array}{c} x_{r}\\ y_{r}\\ \theta_{r} \end{array}\right]}_{k-1}}}+\underset{B_{k}}{\underset{︸}{\left[\begin{array}{cc} \cos(\theta_{r,k-1}) & 0\\ \sin(\theta_{r,k-1}) & 0\\ 0 & 1 \end{array}\right]}}\underset{u_{k}}{\underset{︸}{\left[\begin{array}{c} u_{fwd}\\ u_{ang} \end{array}\right]}},$$

*where the matrix $B_{k}$ describes how the control input affects the state at time k and the $u_{k}$ represents the measured displacement and rotation.*

*In order to refine its position estimate, the robot measures Euclidean distance and relative angle to each of the four landmarks with known positions in the map, see Figure 2b. For a landmark with position $\left(x_{lm},\;y_{lm}\right)$, the measurement model therefore is:*

(4) $${\left[\begin{array}{c} z_{dist}\\ z_{ang} \end{array}\right]}_{k}=\begin{array}{c} \sqrt{{\left(x_{r,k}-x_{lm}\right)}^{2}+{\left(y_{r,k}-y_{lm}\right)}^{2}}\\ \arctan\left(\frac{y_{r,k}-y_{lm}}{x_{r,k}-x_{lm}}\right) \end{array}.$$

*Notice that for reasons of simplicity, the robot’s heading is not considered in the simulated measurement, i.e., both $z_{dist}$ and $z_{ang}$ are independent of the robot’s heading $\theta_{r}$.*

### 3.2. Mathematical Problem Statement

From now on we move to a probabilistic notation, describing the state estimation problem addressed by the particle filter as a Bayesian estimation problem. A probability density function (pdf) over a random variable *a* will be denoted p(a), the conditional probability density function of a random variable *a* given *b* will be denoted p(a∣b).

The state estimate is represented by a pdf that quantifies both the estimated state and the uncertainty associated with the estimated value. The state of interest is referred to as the posterior state: the estimate of the state at time *k* given all measurements and inputs up to time *k* and is denoted by the conditional pdf:

(5) $$p(x_{k}|u_{1:k},z_{1:k}).$$

In (5) $u_{1:k}=\left\{u_{i};\;i=1,\ldots ,k\right\}$ denotes the sequence of known control inputs and $z_{1:k}=\left\{z_{i};\;i=1,\ldots ,k\right\}$ denotes the measurement sequence. In the remainder, control inputs will be left out for ease of notation. The posterior state is the state that will be estimated at each time step.

The state sequence is modelled by a Markov chain. This means the past is assumed to be adequately summarized by only the state at the previous time step. Mathematically this implies:

(6) $$p\left(x_{k}|x_{1:k-1},z_{1:k-1}\right)=p\left(x_{k}|x_{k-1}\right),\;p\left(z_{k}|x_{1:k}\right)=p\left(z_{k}|x_{k}\right).$$

In words, when modeling how the state is expected to change from time k−1 to time *k*, it is assumed that state $x_{k-1}$ implicitly includes all information needed. Older states $x_{1},\ldots ,x_{k-2}$ and previous measurements $z_{1},\ldots ,z_{k-1}$ will therefore be discarded after being processed. Similarly, only control input $u_{k}$ is used for problems including a control input. For measurements a similar reasoning applies, as shown in (6).

The particle filter is a Bayesian filter. This means, estimation is performed using Bayesian theory. Bayesian inference allows for estimating a state by combining a statistical model for a measurement (likelihood) with a prior probability using Bayes’ theorem. Mathematically, Bayes’ theorem can be written as: p(A∣B)=P(B∣A)P(A)/P(B). In words, Bayes’ theorem can be written as:

(7) $$\mathrm{posterior}=\frac{\mathrm{likelihood}\cdot \mathrm{prior}}{\mathrm{marginal}\_\mathrm{likelihood}}.$$

The marginal likelihood acts as normalization term and only depends on measurements. Tracking problems can be solved by recursively applying the predict–update cycle that is common in Bayesian filtering [6]. Step 1 is computing the prior using a process model, step 2 is refining the estimate using Bayes’ theorem.

1. The process model is used to compute the state that is expected at time *k* given all measurements up to time k−1.
2. A measurement $z_{k}$ at time *k* is used to refine the expected state estimate using Bayes’ theorem, leading to the posterior. The prior is computed in step 1 and the likelihood is related to $z_{k}$, as will be explained later. Typically, knowledge on the sensor characteristics of the sensor that generated the measurement is used to compute the likelihood. A more elaborate explanation of Bayes’ theorem can be found in [31].

Mathematically these steps can be written as follows. The posterior distribution at the previous time step, $p(x_{k-1}|z_{1:k-1})$, is combined with the process model that describes how the state evolves over time in the prediction step. The result is referred to as the prior state:

(8) $$p\left(x_{k}|z_{1:k-1}\right)=\int p\left(x_{k}|x_{k-1}\right)p\left(x_{k-1}|z_{1:k-1}\right)dx_{k-1}.$$

The prior represents the best guess at time *k* given measurements up to time k−1. It can be interpreted as the predicted state at time *k*.

During the update step, the measurement $z_{k}$ at time *k* is used to compute the posterior using Bayes’ theorem:

(9) $$p\left(x_{k}|z_{1:k}\right)=\frac{p\left(z_{k}|x_{k}\right)p\left(x_{k}|z_{1:k-1}\right)}{p(z_{k}|z_{1:k-1})}.$$

The likelihood $p(z_{k}|x_{k})$ represents the conditional probability of a measurement given the predicted state, $p(x_{k}|z_{1:k-1})$ is the prior computed using (8) and the normalizing constant represents the probability of the measurement. It can be computed using:

(10) $$p\left(z_{k}|z_{1:k-1}\right)=\int p\left(z_{k}|x_{k}\right)p\left(x_{k}|z_{1:k-1}\right)dx_{k}.$$

Bayesian filters combine prior knowledge on how the state is expected to evolve over time with measurements that include information related to the current state. More elaborate mathematical derivations can be found in [6,11]. In geophysics often the term ‘data assimilation’ is used instead of filtering [17].

Contrary to least squares minimization, where a batch of measurements is processed by finding a parameterized function that best fits that data, Bayesian filters refine the state estimate each time a new measurement is received. Both methods are introduced and compared elaborately in [31].

### 3.3. Joint State Estimation and Model Selection

In some applications, different models must be defined and accurate state estimation requires to jointly estimate the state and the most suitable model. Consider for example a state that represents the position of a person and different process models are defined for describing the person’s next position: one for the person being in a car and one for the person walking. Model selection is excluded from the problem statement in this tutorial. More information can be found in [32,33].

## 4. Particle Filter

This section introduces the particle filter as a method to solve state estimation problems. The idea underlying the filter will be presented together with its generic formulation in Section 4.1. Then, Section 4.2 explains five challenges that should be considered when using particle filters.

### 4.1. Basic Idea

The integrals in (8) and (9) can only be solved analytically under strong assumptions, e.g., for finite dimensional discrete state variables or linear models and Gaussian pdfs. Rather than restricting the models, the particle filter approximates the pdf representing the posterior by a discrete pdf such that there are minimal restrictions on the models involved. The optimal Bayesian solution is approximated by a sum of weighted samples:

(11) $$p\left(x_{0:k}|z_{1:k}\right)\approx \sum_{i=1}^{N_{s}}w_{k}^{i}\delta \left(x_{0:k}-x_{0:k}^{i}\right).$$

Here ${\left\{w_{k}^{i},x_{0:k}^{i}\right\}}_{i=1}^{N_{s}}$ is a set containing $N_{s}$ samples and weights. Each sample $x_{0:k}^{i}$ represents a possible realization of the state sequence. A weight $w_{k}^{i}$ represents the relative importance of each of the $N_{s}$ samples $x_{0:k}^{i}$ and $\sum_{i=1}^{N_{s}}w_{k}^{i}=1$. Samples associated with high weights are believed to be closer to the true state sequence than samples associated with low weights. δ(·) denotes the Dirac delta function. The Dirac delta function δ(a) is zero everywhere except for *a*, its integral is equal to one.

The discrete approximation of a continuous pdf turns intractable integrals into summations over $N_{s}$ samples. The samples are usually referred to as particles hence the name particle filter. The advantages of representing the posterior by a set of weighted particles include (i) the ability to represent arbitrarily shaped pdfs (assuming enough samples) and (ii) minimal restrictions on the process and measurement models. This combination of advantages is one of the main reasons for the popularity of the particle filter. The most obvious drawbacks are (i) the lack of expressiveness in case the number of particles is too low and (ii) the increased computational costs (more on that in Section 4.2).

The sample-based approximation comes with an obvious challenge. The posterior pdf that must be estimated is unknown hence sampling from it is impossible. Samples must therefore be drawn from another distribution instead. This distribution, referred to as importance density (sometimes called proposal density) will be denoted *q*. The weights compensate for the fact that samples are drawn from the importance density *q* rather than the posterior pdf. Any function that is positive where the posterior is positive can be used as importance density.

**Figure 3:**
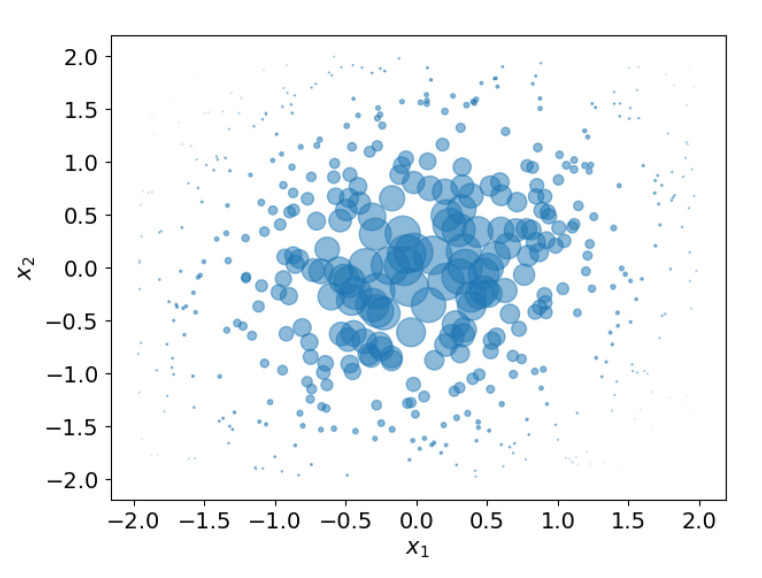
Set of 500 weighted samples (clue circles) estimating a 2D zero mean Gaussian distribution. The radius of a sample is proportional to its weight.

**Example** **2.** *Figure 3 shows an example in which weighted samples from one distribution are used to approximate another distribution. In this example, a zero mean 2D Gaussian distribution must be estimated by a set of samples, i.e., the Gaussian distribution plays the role of posterior pdf. Samples are drawn from a 2D uniform distribution that plays the role of importance density q. Obviously, the samples from the uniform distribution are an inaccurate reflection of the Gaussian distribution. For that reason, weights are used to compensate for the difference between ‘importance density’ and ‘posterior distribution’. With the right weights, the weighted samples provide a reasonable estimate of the to be estimated Gaussian distribution, as is shown in Figure 3.*

The next question is: given the importance density *q* and without knowing the posterior, how can the weights be computed? It can be shown [6,12,14] that the correct way of computing the weights is:

(12) $$w_{k}^{i}\propto \frac{p(x_{0:k}^{i}|z_{1:k})}{q(x_{0:k}^{i}|z_{1:k})}.$$

Here the symbol ∝ means proportional to and the term on the right-hand side is the ratio between posterior and importance density for an individual particle with index *i*.

In recursive filtering, typically the state at time step *k*, $p(x_{k}|z_{1:k})$, is of interest, rather than the full state sequence $p(x_{0:k}|z_{1:k})$ up to time *k*. As a result, (12) can be rewritten:

(13) $$w_{k}^{i}\propto w_{k-1}^{i}\frac{p\left(z_{k}|x_{k}^{i}\right)p\left(x_{k}^{i}|x_{k-1}^{i}\right)}{q(x_{k}^{i}|x_{k-1}^{i},z_{k})},$$

where $w_{k-1}^{i}$ represents the weight at the previous time step for particle *i*. Without further derivations we present the resulting posterior:

(14) $$p\left(x_{k}|z_{1:k}\right)\approx \sum_{i=1}^{N_{s}}w_{k}^{i}\delta \left(x_{k}-x_{k}^{i}\right).$$

By increasing the number of particles, this estimate can be shown to converge to the exact solution [6,12,14]. The same references give mathematical derivations of the equations given above.

In summary, when running the algorithm described so far, the sequential importance sampling (SIS) algorithm is obtained. SIS is described by the pseudo code of Algorithm 1.
**Algorithm 1:** Sequential Importance Sampling
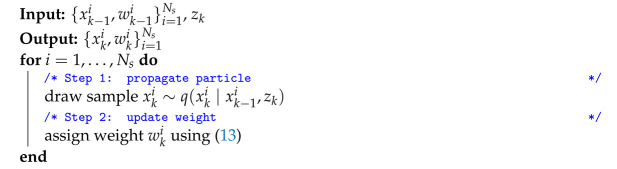

**Example** **3.** *For the robot localization example, both the process and measurement noise are assumed to be zero mean Gaussian distributions with diagonal covariance matrices Q and R respectively. The fact that covariance matrices are diagonal represents uncorrelated noise in each dimension. The prediction and update steps can be explained as follows.*

*The most common choice for the importance density is $p(x_{k}^{i}|x_{k-1}^{i})$ hence this importance density will be used for the running example as well. As a result:*

(15) $$x_{k}^{i}∼N\left(x_{k-1}^{i}+B_{k}u_{k},Q\right),$$

*In words: during the prediction step the particle state is obtained by drawing a sample from a Gaussian distribution with mean vector $x_{k-1}^{i}+B_{k}u_{k}$ and covariance matrix Q. The mean of the Gaussian distribution is computed by applying the process model (3) to particle i at time k−1. The covariance Q of the Gaussian distribution is the 3×3 covariance matrix of the additive zero mean process model noise v. The fact that a sample is drawn means this step is stochastic.*

*The second step is updating the weights. Since the importance density is selected to be $p(x_{k}^{i}|x_{k-1}^{i})$, (13) simplifies to [6]:*

(16) $$w_{k}^{i}\propto w_{k-1}^{i}p\left(z_{k}|x_{k}^{i}\right),$$

*where weight $w_{k-1}^{i}$ is known from the previous time step. The expected measurements assuming $x_{k}^{i}$ and using the measurement model (4) are denoted:*

$${\left[\begin{array}{c} {\hat{z}}_{dist}^{i}\\ {\hat{z}}_{ang}^{i} \end{array}\right]}_{k}^{j},$$

*where j=1,2,3,4 denotes the landmark index, k the discrete time step and i the particle index. The measurement noise $n_{k}$ is assumed to be additive zero mean Gaussian noise with a 2×2 covariance matrix R and the likelihood is computed using (assuming four independent landmark measurements):*

(17) $$p\left(z_{k}|x_{k}^{i}\right)=\prod_{j=1}^{4}N\left({\left[\begin{array}{c} {\hat{z}}_{dist}^{i}\\ {\hat{z}}_{ang}^{i} \end{array}\right]}_{k}^{j};{\left[\begin{array}{c} z_{dist}\\ z_{ang} \end{array}\right]}_{k}^{j},R\right),$$

*where the second and third term in $N\left(\cdot ;\cdot ,\cdot \right)$ represent the mean vector and covariance matrix of a Gaussian distribution. The first term represents the point at which this Gaussian distribution must be evaluated. In words: a 2D Gaussian distribution with its mean vector equal to the actual measurement and a covariance matrix equal to the measurement noise covariance will be evaluated at the location of the expected measurement. The highest weight possible is obtained in case the expected measurement coincides with the actual measurement. The larger the difference between expected and actual measurement, the lower the likelihood (17) that will be used to update the particle weight in (16). Weights will be normalized to ensure that all weights sum up to one. Particle states $x_{k}^{i}$ are not changed during this step and the computation of weights is deterministic.*

*This completes the explanation of the implementation of the basic particle filter as it is used in the running example. The scriptdemo_running_example.pydemonstrates how the particle filter performs for this simulation setup. The two steps above are implemented in theupdatefunction ofparticle_filter_sir.py*.

**Figure 4:**
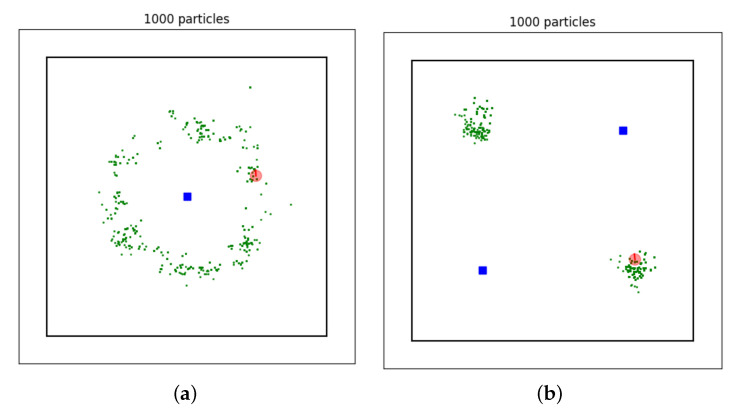
Simulation environments with different numbers of landmarks (blue rectangles). The range only measurements do not provide sufficient information for uniquely finding the true robot pose (red). The particles (green dots) converge to a more complicated posterior distributions. (**a**) One landmark: donut-like shaped posterior. (**b**) Two landmarks: bimodal distribution.

**Example** **4.** *One of reasons for the popularity of particle filters is their ability to represent posterior distributions of arbitrary shape. In order to demonstrate this, the running example is slightly modified. Only the distance to landmarks is measured and the number of landmarks and their positions is varied. As a result the particles form complicated, non-Gaussian posterior distributions: a donut-like shape and a bimodal distribution, see Figure 4. These posterior distributions can be reproduced by changing the settings in* *demo_range_only.py* *. Real world applications adopting a particle filter for robot localization in a similar setup include [34,35].*

### 4.2. Particle Filter Challenges

This section explains five challenges that must be addressed before designing and implementing a particle filter.

#### 4.2.1. Challenge I: Degeneracy Problem

**Figure 5:**
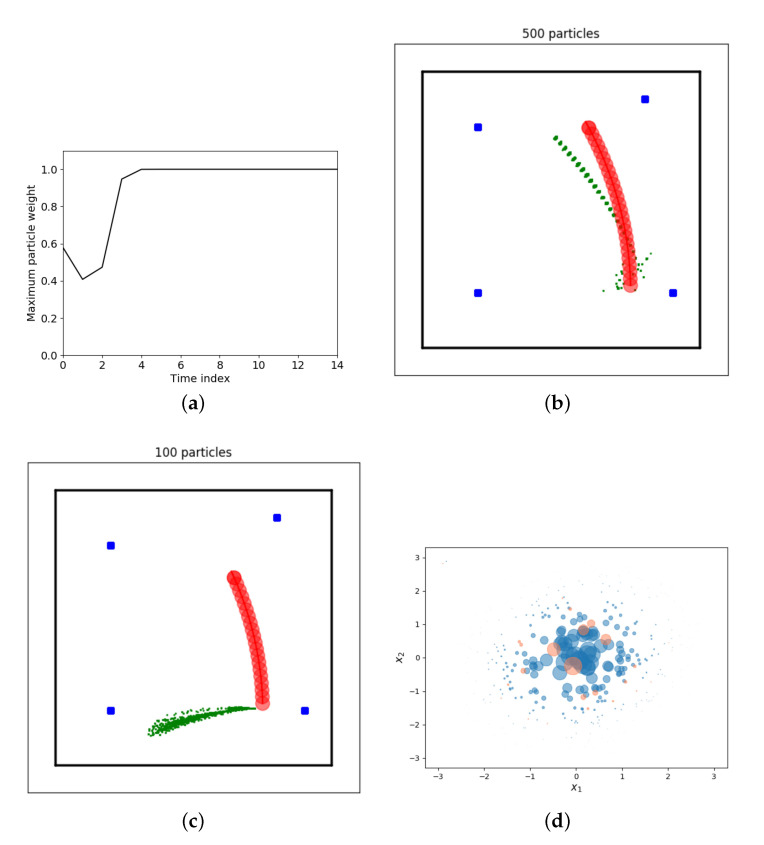
Visualizations of four out of the five challenges identified for particle filters. (**a**) Challenge I: degeneracy. (**b**) Challenge II: sample impoverishment. Red: robot trajectory, blue: landmarks, green: particles at different time steps. (**c**) Challenge III: particle filter divergence. Red: robot trajectory, blue: landmarks, green: particles at different time steps. (**d**) Challenge IV: selecting the importance density.

After a few iterations one particle weight will be very close to one and all other particle weights will be almost zero in case Algorithm 1 is used. This problem is known as degeneracy problem and it cannot be avoided [36]. An example of the maximum normalized particle weight for a simulation with our running example is provided in Figure 5a. After the first update step (time index zero in the plot) roughly half of the total weight originates from a single particle. From time step five onward, the maximum weight is one and the remaining 499 particles have a negligible contribution. This figure is generated by running challenge1_degeneracy.py. The precise shape of the curve will change for each run due to the stochastic nature of the particle filter and the simulated robot motions and measurements.

The consequences of the degeneracy problem are (i) almost all computational effort will be put into computations related to particles that have negligible or no contribution to the overall estimate and (ii) the number of effective particles is only one. The latter greatly limits the performance and expressiveness of the filter. The expressiveness is limited since a single particle can only represent one point in the state space rather than pdfs of arbitrary shapes. Performance is poor since the particle filter will diverge. Divergence occurs in case state estimation errors increase over time and are unacceptably large. Once diverged, the filter fails to ‘follow’ the true state or reduce estimation errors to acceptable values. An example of a diverging estimate can be found in Figure 5c.

The solution to this problem is resampling. Resampling is performed directly after the update step. In the resampling step, new particles are randomly selected, with replacement, from the set of weighted particles. The probability of selecting a particle is proportional to its weight and the number of particles remains unchanged. Particles with higher weights are likely to be duplicated, whereas low weight particles are likely to be removed. After resampling the weights are typically reset to $1/N_{s}$.

**Figure 6:**
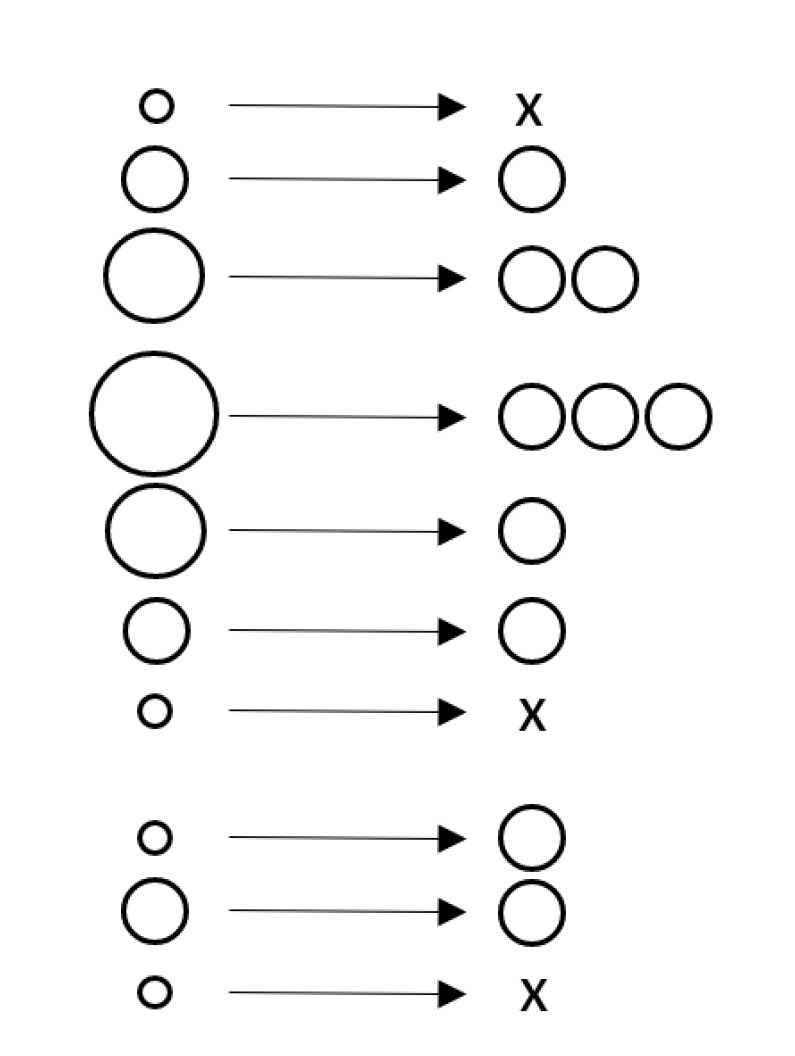
Example of how resampling changes a set of 10 weighted particles (**left**) into a new set of 10 uniformly weighted particles (**right**). Some particles are duplicated, some particles are never selected and disappear (represented by the ’x’).

**Example** **5.** *Resampling is schematically visualized in Figure 6. The weighted particles for a 1D state are shown on the left, where the size reflects the weight. During resampling, some particles are selected multiple times whereas other particles are not selected at all as is indicated by the ‘x’. Notice the resampling step is stochastic: if this step is repeated multiple times with the same set of weighted particles, the results will be similar but different.*

Different resampling schemes (when to perform resampling), and resampling algorithms (how to resample) will be thoroughly explained and demonstrated in Section 5.1.

#### 4.2.2. Challenge II: Sample Impoverishment

Solving the degeneracy problem by resampling leads to multiple instances of the same particle, as can be seen in Figure 6. If we assume the most popular choice for the importance density, $p(x_{k}^{i}|x_{k-1}^{i})$, the prediction step contains a deterministic and a stochastic part (we draw a sample from a pdf). As a result, particles with the same state will diversify during the prediction step. If the process model noise variance is very low, samples will hardly diversify and after a few iterations all particles will collapse into a single point in the state space. Then, all $N_{s}$ particles will represent the same state and again the filter effectively only has one particle. This problem is referred to as sample impoverishment. Like with particle degeneracy, the filter will lose performance, expressiveness and will likely diverge.

Figure 5b demonstrates the problem of sample impoverishment for our running example. The red circles represent the robot trajectory to be estimated, the blue rectangles are landmarks used for positioning the robot and the green dots represent all $N_{s}$ particles at each of the time steps. The dotted appearance of the particles is the result of sample impoverishment. All 500 particles at each time step are very close to each other and appear as a single ‘dot’ in the plot. Due to limited noise in the propagation step, the cloud does not diversify and effectively the number of particles becomes close to one. This figure can be reproduced by executing challenge2_impoverishment.py. Again, the precise appearance may change due to the stochastic nature of both filter and simulation.

In general, a sufficient amount of process model noise prevents sample impoverishment, however, many works have further investigated sample impoverishment. Later sections will thoroughly investigate and demonstrate the most popular solutions.

#### 4.2.3. Challenge III: Particle Filter Divergence

When employing a particle filter for a real-world problem, the risk of particle filter divergence must always be considered. A diverged filter is not able to provide accurate estimates and therefore is problematic for the application at hand. Reasons for divergence can range from poor tuning of the filter or incorrect modeling assumptions to inconsistent measurement data or hardware failures. Examples include incorrect or inaccurate measurement noise assumptions while computing likelihoods, inaccurate process models or latency in the transmission of a measurement to the particle filter algorithm. Figure 5c shows a clear example of particle filter divergence: all particle have unacceptably large estimation errors and the errors increase over time. This figure is generated using by running challenge3_divergence.py.

Particle divergence monitoring is an inevitable part of any particle filter that is expected to deliver estimates for real world problems in real time.

#### 4.2.4. Challenge IV: Selecting the Importance Density

In Section 4.1, the role of the importance density was explained. This function is at the core of the particle filter algorithm and for that reason selecting a proper function *q* is one of the most important steps in the design of a particle filter. In [37], it is stated that ‘when designing a particle filter for a particular application, it is the choice of importance density that is critical’. In practice, the number of candidates is limited, as will be explained in Section 5.4.

Figure 5d illustrates the role of the importance density. The posterior that must be estimated is a zero mean Gaussian distribution and the importance density is a uniform distribution. This choice of importance density is valid but not very informative. Consequence is that a relatively high number of particles is required to capture the shape of the underlying distribution: with 500 weighted samples the Gaussian distribution is represented reasonably well (blue circles). With 50 weighted samples, only a few samples are within the relevant part of the state space (red samples). Although Figure 5d does not exactly mimic the particle filter setting, it should help in understanding how a poor importance density greatly complicates getting good state estimation performance using a particle filter.

#### 4.2.5. Challenge V: Real Time Execution

A particle filter can only work if the number of particles is sufficient to represent the distributions that are being estimated. As a result, the dimension of the state space has a big impact on the number of particles needed. At the same time the number of particles directly affects the computational costs of the filter. The fifth challenge that will be elaborated on are aspects related to real time execution of the particle filter algorithm.

## 5. Solutions to the Particle Filter Challenges

This work aims at providing a complete picture of all five challenges introduced in Section 4, as a practitioner interested in getting familiar and implementing the particle filter is likely to encounter all of them. The goal of this section is enabling the selection of a suitable method for a specific estimation problem the reader needs to tackle. The code accompanying the paper implements at least one of the solutions for each of the challenges and references to the code are given when applicable. Experimenting with both the challenge and solution is highly encouraged since this helps understanding the problem and getting acquainted with the solution. To complement the topics we provide further references for the readers interested in details specific to each challenge.

### 5.1. Challenge I: Degeneracy Problem

In this section resampling algorithms will be presented in more detail. As explained in Section 4, resampling prevents the degeneracy problem and is therefore crucial in any particle filter. Throughout this work, algorithms that determine when to resample are referred to as ‘resampling schemes’. Algorithms doing the actual resampling are referred to as ‘resampling algorithms’.

#### 5.1.1. Resampling Schemes

The simplest resampling scheme possible is resampling at every time step, as is explained in Algorithm 2.
**Algorithm 2:** Particle filter with resampling
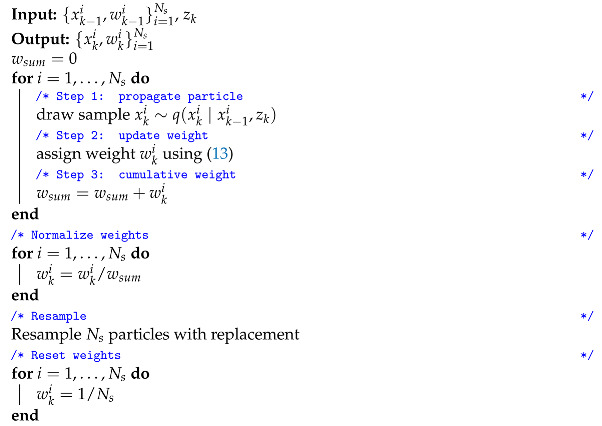


This strategy avoids the degeneracy problem and is among the most popular solutions. One aspect to keep in mind when adopting this solution is computational cost. Resampling is costly in terms of computation and resampling at every time step is a conservative way to avoid particle degeneracy. In other words: particle degeneracy could as well be prevented by resampling less frequently. Furthermore resampling at every time step usually reduces the diversity in the particle set which may increase the risk of sample impoverishment (challenge II).

It would suffice to resample whenever the particle weights indicate some form of particle degeneracy. The most common way to assess particle degeneracy is by evaluating the effective sample size, introduced in [38]. It can be approximated by (see [39] for more details):

(18) $${\hat{N}}_{eff}=\frac{1}{\sum_{i=1}^{N_{s}}{\left(w_{k}^{i}\right)}^{2}}.$$

Algorithm 3 summarizes the particle filter algorithm with resampling based on ${\hat{N}}_{eff}$ in pseudo code. Loosely formulated this means that resampling happens whenever the summed weights of a relatively small (compared to $N_{s}$) subset of particles is responsible for a large part of the total weight.

Most particle filters use one of the two resampling schemes presented above, especially for lower dimensional states. However, many other measures can be used to quantify the effective number of samples. One could for example decide to measure the effective sample size by $1/\max_{i}\left(w_{k}^{i}\right)$ and resample if this number drops below a user defined threshold. This would only require changing line 8 in Algorithm 3. An elaborate overview and comparison of effective sample size measures can be found in [40]. The code accompanying this paper implements the three options explained in this section: ’every time step‘-resampling is implemented in particle_filter_sir.py, resampling based on the approximate number of effective particles is implemented in particle_filter_nepr.py, resampling based on the maximum particle weight criterion is implemented in particle_filter_max_weight_resampling.py.
**Algorithm 3:** Particle filter with resampling threshold
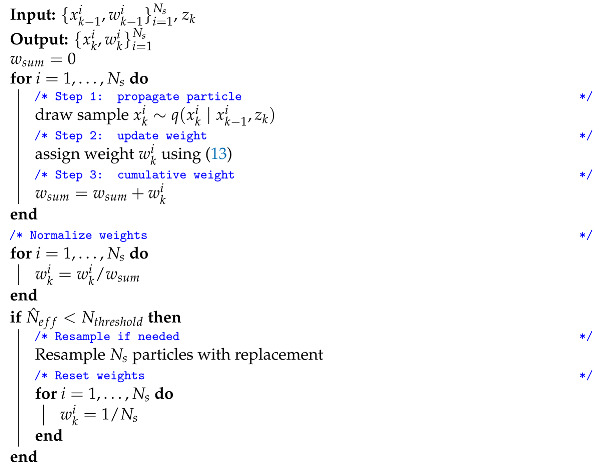


In case none of the solutions above is sufficient, less common resampling schemes can be adopted. Consider for example [41], where the resampling scheme and algorithm are combined. Resampling is based on some criterion, e.g., only those particles that have weights below a predefined value. Furthermore, sampling with replacement is replaced by regenerating particles in specific regions of the state space (defined based on domain specific expert knowledge). Another approach is deterministic resampling [42]. With deterministic resampling both the weight and the state of a particle are considered when resampling, thereby avoiding ‘uncensored discarding of low weighted particles’. Regularized particle filters resample from a continuous approximation of the posterior rather than using (11) [6]. This typically is done by replacing the Dirac delta function by a kernel density. A kernel is a symmetric pdf and is a function of the particle state. In [6], the optimal kernel is given in the special case $w_{i}=1/N_{S}$ for all particles.

**Example** **6.** *The running example is used to compare three resampling schemes:*

*1*. *Resample every time step (Algorithm 2).*
*2*. *Resample if ${\hat{N}}_{eff}<N_{s}/4.0$ (Algorithm 3).*
*3*. *Resample if $1/\max_{i}\left(w_{k}^{i}\right)<1/0.005$.*

The simulation has 50 time steps and is repeated 100 times. The mean and standard deviation of the 2-norm of the error vector over all 100*50 time steps are computed. The results are summarized in Table 1. The script associated with these simulations is *challenge1_compare_resampling_ schemes.py*

*The thresholds are selected without much tuning and therefore, the results are indicative and will mainly be used for showing typical differences. In fact, the results will change if this simulation would be repeated, due to the stochastic nature of the algorithm. The resampling scheme has some effect on the mean errors for this simulation (order of a few percent) whereas the standard deviations are very similar. The advantage of using a more advanced resampling scheme becomes evident in the last column. The number of resampling steps is 3.9 times lower for the resampling scheme that uses the approximate estimated number of effective particles and 2.8 times lower for the resampling scheme that monitors the maximum particle weight.*

#### 5.1.2. Resampling Algorithms

Resampling is a stochastic step that turns a set of $N_{s}$ weighted particles into a new set of $N_{s}$ (usually uniformly) weighted particles. Methods that change the number of particles on the fly will be presented in Section 5.5, here it is assumed that the number of particles is constant.

Although the number of existing resampling algorithms is large, most particle filters use one of the following algorithms: multinomial resampling, residual resampling, systematic resampling or stratified resampling. The code accompanying this paper implements all four algorithms and can be used to investigate practical differences among them (see resampler.py). Pseudo code or detailed explanations of the algorithms itself can be found in the overview paper [25]. Refs. [25,43] also compare some relevant properties such as computational complexity and the variance in the number of times particles are selected.

In [26], an effort is made to determine which of the four algorithms performs best. Based on reasoning, residual and stratified sampling are said to outperform multinomial resampling. Similarly, it is argued that systematic resampling cannot be shown to consistently outperform multinomial resampling. Contrary to the results in [26], systematic resampling is concluded to be favorable both in terms of resampling quality and computational complexity in [27].

In general, there is no best resampling algorithm: different papers draw conflicting conclusions as explained above. In case one must decide which one of these algorithms to use it is recommended to at least be aware of the difference in ‘predictability’: when running one resampling algorithm multiple times on the same set of weighted particles, the variance in the number of times each particle is selected by the resampling algorithms varies greatly. Furthermore, some methods may lead to biased results, as is explained and demonstrated in [43].

**Example** **7.** *To give an idea about the differences in outcome when using one of the four resampling algorithms a random set of five weighted particles has been generated. The weights were 0.366, 0.354, 0.119, 0.058, 0.102, and each resampling algorithm has been run 100,000 times. The number of times each particle has been selected is counted for each of these runs. Table 2 and Table 3 show (i) the mean number of times each particle has been selected and (ii) the standard deviation in the number of times each particle has been selected. The goal of this example is to illustrate differences between the algorithms, not to determine which method performs best.*

*The probability of selecting a particle is proportional to its weight and as a result the average number of times each particle is selected is the same for all resampling strategies. The standard deviation measures the variation in the number of times each particle has been selected. This is one of the aspects on which the resampling algorithms differ significantly as can be seen in Table 3. Readers that would like to understand where these differences come from are recommended to study the algorithmic differences reported in [25]. The script associated with this simulation is* *challenge1_compare_resampling_algorithm.py*

### 5.2. Challenge II: Sample Impoverishment

There are various ways to handle the risk of sample impoverishment. This section explains various options that vary in complexity and together represent the vast majority of solutions found in practice. More options can be found in [14,44].

#### 5.2.1. Roughening

The first solution that could come into mind when thinking of ways to avoid sample impoverishment is artificially increasing the process model noise to a level that prevents impoverishment [1]. The work of [28] explains two pragmatic methods that do so and are referred to as ‘roughening methods’.

1. Adding artificial noise after resampling.
2. Adding jitter to the process model used in the propagation step (‘direct-roughening’).

Furthermore, it explores different choices related to when applying roughening and to which element of the algorithm: (i) apply at all or some time steps, (ii) apply to all or some particles, (iii) apply to full vector of some parts of it. When to apply roughening, how much noise must be added and to which dimensions is difficult to state in general [28]. It is, however, good to keep the following in mind.

- Whenever sample impoverishment plays a role for a specific problem, roughening can be an effective measure.
- Roughening is more useful in case the number of particles is relatively low.
- The complexity of roughening methods can be varied depending on the needs, this is typically part of the particle filter tuning process.

**Example** **8.** *Due to the amount of noise in the process model associated with the running example, sample impoverishment does not play a role. In fact, for generating Figure 5b, the process noise was artificially lowered by roughly a factor twenty. For this reason and due to the simplicity of implementing the concept it is not part of the code examples.*

*Roughening can usually be added by adding a single line to Algorithms 1–3.*

#### 5.2.2. Auxiliary Particle Filters

The auxiliary particle filter was introduced in [45]. More details on auxiliary particle filters and derivations can be found in [6] or [14]. Here, the resulting algorithm and the underlying ideas will be explained.

In the standard particle filter (Algorithm 2), step one (prediction) is to randomly draw samples and step two is updating weights using a measurement and the predicted particle states from step one (see Algorithm 2). The rational of auxiliary particle filters is to, in the prediction step, favor particles that are likely to get high likelihoods after incorporating the measurement in the update step. In order to do so, the availability of the latest measurement is exploited in the prediction step, rather than blindly drawing samples from the prior [45]. It uses ‘resampling on predicted particles to select which particles to use in the prediction and measurement update’ [46]. For implementation details, the reader is referred to the code: auxiliary_particle_filter.py. The steps below summarize the auxiliary particle filter algorithm.

1. Compute $N_{s}$ point estimates that are used to characterize $p(x_{k}|x_{k-1}^{i})$: $\mu_{k}^{i}∼p\left(x_{k}|x_{k-1}^{i}\right)$. Different characterizations are possible leading to different modifications of the particle filter. The code accompanying the paper implements the version explained here, see [6,45,46] for more information. Then compute weights for these characterizations: $w_{k}^{i}\propto p\left(z_{k}|\mu_{k}^{i}\right)w_{k-1}^{i}$. Afterwards, normalize the weights.
2. Use weights $w_{k}^{i}$ from step 1 in a resampling step. During resampling, store the indices $i^{j}$ of the particles that would have been selected but do not perform the actual resampling, i.e., do not duplicate or delete particles. Each index $i^{j}$ refers to a particle at time k−1 and the set of indices represents the set of particles that are expected to get high likelihoods.
  $N_{s}$ indices $i^{j}$ are stored during this resampling step. Some particle indices appear multiple times whereas others are not selected at all.
3. Perform a prediction step for each of the $N_{s}$ particle indices $i^{j}$ from step 2.
4. Compute the weights for the propagated particles from step three using:
  (19) $$w_{k}^{j}=\frac{p(z_{k}|x_{k}^{j})}{p(z_{k}|\mu_{k}^{i^{j}})},$$
  where the denominator is the likelihood of the characterization computed in step 1 and the nominator is the likelihood of the propagated particle from step 3. The likelihoods $p(z_{k}|x_{k}^{j})$ are expected to be high due to steps 1 and 2.
  Normalize afterwards.

In the algorithm described above the characterizations are used to predict whether a particle is expected to get a high likelihood given the measurement. Whenever two characterizations of the same state may differ significantly, the likelihoods after incorporating the measurement may also differ significantly and the particle filter may perform worse than the original particle filter. Typically, this is the case for process models with large amounts of noise. In such cases, a single point is not sufficient to characterize the density $p(x_{k}|x_{k-1}^{i})$ and an auxiliary particle filter performs worse than a standard particle filter. In general, the auxiliary particle filter outperforms the standard particle filter [6], however, the number of likelihoods that must be computed is $N_{s}$ for the standard particle filter and $2N_{s}$ for the auxiliary particle filter. This may complicate real time execution with many particles, especially if computing likelihoods is computationally expensive.

**Figure 7:**
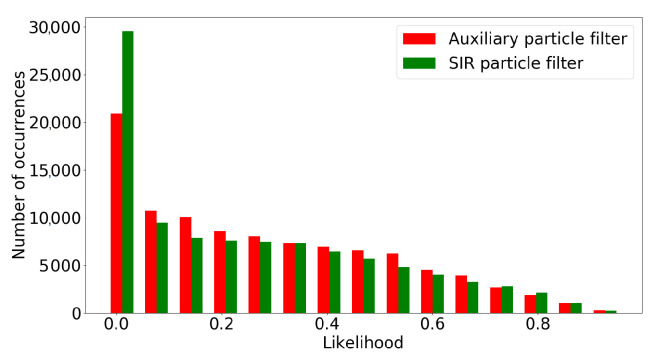
Distribution for the likelihoods of the auxiliary particle filter and the standard particle filter.

**Example** **9.** This example aims at showing the effect of favoring particles that are likely to get high likelihoods, as is done in auxiliary particle filtering. In order to do so, both the standard particle filter (Algorithm 2) and the auxiliary particle filter are simulated 100 time steps with 1000 particles. Next, we plot the distribution of the 100*1000 likelihoods $p(z_{k}|x_{k}^{j})$ for both filters. The relation between likelihood and particle weight has been shown in (13) and (19). Figure 7 shows the likelihoods and confirms that the auxiliary particle filter indeed leads to higher likelihoods.

#### 5.2.3. Resample Move Step

A more refined way of preventing impoverishment is implementing a resample move step. Here, the Metropolis–Hastings (MH) step will be explained, but different variants exist [44]. The MH step is added after resampling and increases the variety in particles by either keeping a resampled particle, or replacing it by a new particle that is obtained by repeating the predict and update steps as explained in Algorithm 4.
**Algorithm 4:** Metropolis–Hastings step
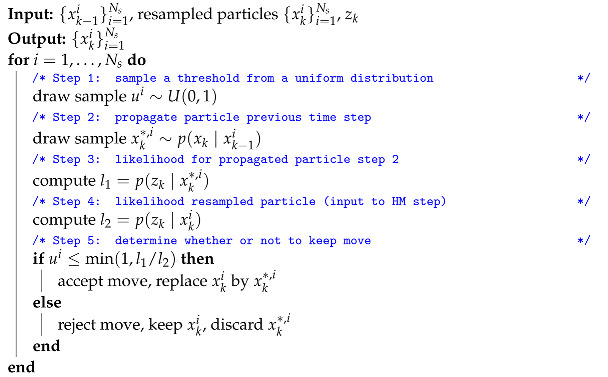


#### 5.2.4. Further Ways to Minimize Particle Degeneracy and Impoverishment

A more recent approach that aims for keeping particles in regions of high likelihood includes [47], where a deep belief network and particle swarm optimization are combined in what is referred to as deep-belief-network-based particle filter. A more elaborate review of different methods, including the auxiliary particle filter explained in Section 5.2.2, the regularized particle filter explained in Section 5.1.1 and data-driven and artificial intelligence-based methods can be found in [30].

### 5.3. Challenge III: Particle Filter Divergence

Whenever state estimation is performed recursively, the estimates may diverge. A particle filter has diverged whenever the particles no longer reflect the true state. Without an explicit mechanism to handle particle filter divergence it is not possible to ensure reliable estimates in an online setting. A list of major causes of particle filter divergence is given below.

- A process model that only covers a subset of all possible state transitions. In case the true state dynamics are not part of the process model none of the particles get propagated into the part of the state space that most accurately describes the true state.
- Measurement noise is modeled much lower than the actual noise level. Measurements that are realistic given the high amount of measurement noise get very low likelihoods: the measurement model judges such amounts of noise as highly unlikely and the associated particles will be removed during the resampling step.
- The usage of incorrect measurements, e.g., unwanted reflections caught by a radar, outliers; it leads to fusing incorrect information in the estimates.

The easiest way to recover from divergence is reinitializing the particle filter. Obvious prerequisite is a way to detect particle filter divergence. Although the literature on this topic is rare, some approaches are available.

One way to monitor particle filter divergence is by computing the approximate number of effective particles using (18). The most popular approach uses unnormalized likelihoods instead [14]. Whenever all likelihoods are low, the predictions fail to represent the measurement. Both standard hypothesis tests and predefined values can be used for testing whether reinitialization of the filter is needed. Although simple, this method often is sufficiently effective for recovering from particle filter divergence.

The Kullback–Leibler divergence, also known as relative entropy, is a measure of how one probability distribution differs from another. It can be used to determine the similarity between measurement and estimate [31]. Whenever the dissimilarity is too large, the particle filter is reinitialized. A way to compute the Kullback–Leibler divergence for particle filters and the definition of a threshold used to determine whether reinitialization is needed are both explained in [48].

In [49], the difference between the expected and the actual measurement is estimated together with the variance associated with this difference. This difference is fed to a change detection algorithm that performs divergence detection using a sequential test.

Approaches to mitigate particle filter divergence that are tailored to a specific application include [50] for indoor localization using an inertial measurement unit or [51] for pedestrian tracking using inertial navigation system and ultra-wideband technology.

**Example** **10.** *In the simulation set-up accompanying this paper, the sum of all particle weights is computed before normalization. In case this sum is close to zero a warning is printed (see* *particle_filter_base.py* *). The easiest way to explore particle filter divergence is by running* *challenge3_divergence.py* *. This script was used to generate Figure 5c. Adding the reinitialization step should be straightforward and is left as an exercise.*

### 5.4. Challenge IV: Importance Densities

In Section 4.2.4, it was already explained that the choice for an importance density is crucial when designing a particle filter. The prior $p(x_{k}|x_{k}^{i})$ is adopted as importance density in our running example, all particle filters presented so far and the pioneering work of [1]. In fact, the prior is by far the most popular importance density in the literature and is expected to be a suitable choice in most applications [14].

Whenever the predicted particle state contains more information about the next state then the likelihood, using the prior as importance density is a natural choice. This is the case for the overwhelming majority of applications found in the literature. The most popular alternative is to sample from an importance density that is related to the likelihood. Instead of sampling from the prior and then weighting according to the likelihood, samples can be drawn from the likelihood and weights are assigned based on the process model [6]. From the perspective of the challenges explained so far, not much changes within the particle filter. Various likelihood-based importance densities including mathematical derivations and the equation that must be used to compute weights in that case can be found in [6] or [14]. A practical example along this line of thought is explored in [52] Section 3.

The remainder of this section explains two alternative approaches.

#### 5.4.1. Extended Kalman Particle Filter

The Kalman filter is a Bayesian filter that provides the optimal solution for estimation problems where the posterior is a Gaussian distribution, the models involved are linear and the noise in those models is Gaussian with known parameters [6]. In this linear/Gaussian case, the Kalman filter delivers the optimal solution. For non-linear process and/or measurement models approximate Kalman filter solution exists. The first one relies on estimating the nonlinear models by a local linearization, i.e., first order Taylor expansions around the estimated state. This solution is referred to as the extended Kalman filter (EKF).

The EKF can be combined with a particle filter in an effort to construct improved importance densities. In the extended Kalman particle filter, each particle represents an EKF rather than a possible value of the state. The Gaussian posterior distribution of this EKF is adopted as importance density, i.e., a sample will be drawn from the Gaussian distribution. The rational is to approximate the optimal importance density by ‘incorporating the most current observation with the optimal Gaussian approximation of the state’ [44,53]. By changing the importance density, (13) can no longer be simplified to (16). Incorporating EKFs into particles increases the computational costs and poses limitations on the models involved.

The main differences between the extended Kalman particle filter and the standard particle filter, explained in Algorithm 2, are summarized in the list below.

- Each particle is associated with a Gaussian distribution (mean vector and covariance matrix), rather than only a possible value of the state.
- For each particle, an EKF prediction and update step [6] are performed. Result is an updated Gaussian distribution in which the most recent measurement is incorporated. Therefore, the prior is moved towards the likelihood.
- The new particle state vector is sampled from the EKF posterior.

The only way to find out whether or not turning a particle filter into an extended Kalman particle filter leads to superior performance is experimentation. Guaranteeing an improved estimation accuracy is not possible. In fact, it depends on the accuracy of ‘the Gaussian assumption on the form of the posterior’ (which in general is not Gaussian) and the inaccuracies introduced by the linearization [44]. An example in which the extended Kalman particle filter has been successfully combined with the resample-move step explained in Section 5.2.3 is presented in [54].

For the running example, the standard particle filter diverged with 100 particles, whereas the extended Kalman particle filter is able to estimate the location of the robot with this number of particles, see demo_running_example_extended_Kalman_particle_filter.py. Readers interested in the details of the filter are encouraged to study [53] or the code associated with this example.

#### 5.4.2. Unscented Particle Filter

The unscented Kalman filter (UKF) is a second approximate Kalman filter solution that facilitates using Kalman filters for estimation problems with nonlinear models. Compared the EKF, it is able to deliver more accurate and robust estimates [44]. This is the result of using the unscented transform rather than a first order Taylor approximation of the nonlinear models. More details on UKFs and the difference with EKFs can be found in [6].

The unscented particle filter can be implemented by replacing the EKFs in the extended Kalman particle filter by UKFs and has been shown to outperform the extended Kalman particle filter in various simulation examples in [44]. Like with the extended Kalman particle filter, there is no guarantee that the unscented particle filter outperforms the standard particle filter.

### 5.5. Challenge V: Real Time Execution

This section explains ways to handle two important aspects that require consideration when applying particle filters in real time. The first one relates to computational costs of the particle filter, the second one to the order in which measurements are received by the particle filter.

The number of particles needed to approximate a state depends on the dimension of the state space. In fact, populating the state space for a state with dimension *n* requires the number of particles to grow exponentially with *n* [4]. Real time execution of particle filters limits the allowable computational costs and therefore the number of particles. Handling this ‘curse of dimensionality’ can be done in various ways as will be explained in this section.

Real time execution of particle filter may be complicated by out-of-sequence measurements (OOSMs). OOSMs are measurements whose order of measuring is different from the order in which measurements are processed by the particle filter. This typically happens in setups with multiple sensors where transmission times or sensor data acquisition and processing times vary. Ways to handle OOSMs are explained in this section as well.

#### 5.5.1. Rao–Blackwellized Particle Filter

One way to handle the curse of dimensionality is by adopting a Rao–Blackwellized particle filter. Imagine the state sequence $x_{1:k}$ gets partitioned into two smaller parts: $x_{1:k}^{1}$ and $x_{1:k}^{2}$. Now the posterior $p\left(x_{1:k}|z_{1:k}\right)=p\left(x_{1:k}^{1},x_{1:k}^{2}|z_{1:k}\right)$ can be factorized as follows [31]:

(20) $$p\left(x_{1:k}^{1},x_{1:k}^{2}|z_{1:k}\right)=p\left(x_{1:k}^{1}|z_{1:k}\right)\cdot p\left(x_{1:k}^{2}|x_{1:k}^{1},z_{1:k}\right).$$

**Figure 8:**
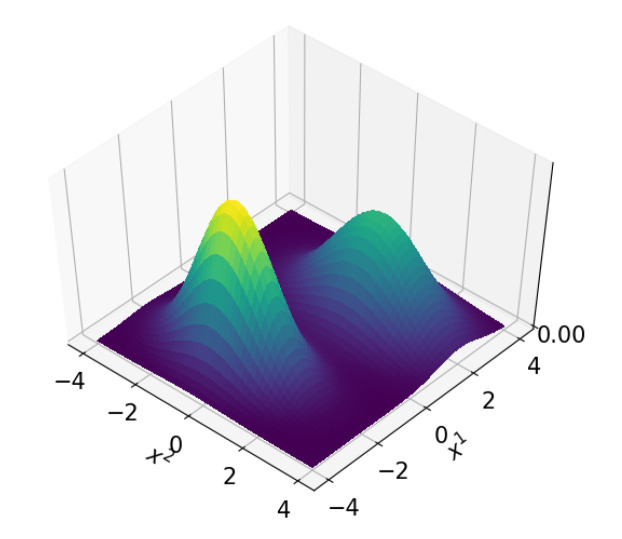
A non-Gaussian pdf that is conditionally Gaussian. More specifically, when knowing $x^{1}$, $x^{2}$ can be approximated by a Gaussian pdf. Notice $x^{1}$ can be a bi-modal, non-Gaussian distribution if $x^{2}$ is given, e.g., for $x^{2}=0$.

In Rao–Blackwellization, a structure of conditional independencies that is present in many estimation problems is exploited for lowering computational costs of the filter. In other words, once the first term on the right-hand side of (20) is known, it is easier to compute the second term. It could for example be that the second term on the right-hand side of (20) conditionally has linear models and Gaussian distributions, in which case the integrals involved can be computed analytically [36]. In that case, usually a Kalman filter is adopted for estimating $p(x_{1:k}^{2}|x_{1:k}^{1},z_{1:k})$ whereas the particle filter estimates $p(x_{1:k}^{1}|z_{1:k})$. Therefore, in a Rao–Blackwellized particle filter, each particle estimates $x^{1}$ and has a Kalman filter that estimates $x^{2}$ assuming the particle’s value for $x^{1}$. A visual example of a non-Gaussian pdf over two variables $x^{1}$ and $x^{2}$, that is Gaussian in case $x^{1}$ is given, is shown in Figure 8.

The Rao–Blackwellized particle filter differs from the Kalman particle filters explained in Section 5.4, where each particle was represented by a Kalman filter that estimates the entire state *x* and no partitioning occurs.

This principle of the factorization in (20) can be generalized for states that are partitioned into more than two parts and for other estimators then the Kalman filter. Rao–Blackwellized particle filter aim at only using particles where needed. For more mathematical backgrounds, the reader is referred to [36]. For an application-oriented explanation, the reader is referred to [55]. Successful applications of Rao–Blackwellized particle filters can be found in, e.g., [56] or [57].

#### 5.5.2. Adaptive Particle Filter

Another way to handle the curse of dimensionality is to vary the number of particles on the fly. Although varying the number of particles does not improve scalability, it is a way to ensure the number of particles is kept as low as possible. The number of particles directly affects the computational costs of the algorithm. The most popular way to do so is proposed by [58]. The idea is to use a small number of particles in case the particles are concentrated on a small part of the state space and an increased number of particles in case the particle filter uncertainty increases. The number of particles is updated such that with predefined probability the error between the true posterior and the sampled-based approximation at every time step is less than ϵ. To avoid the need for more mathematical details, an explanation in words is preferred over pseudo code. For implementation details, the reader is referred to the code (adaptive_particle_filter_kld.py).

1. Sample a particle index proportional to its weight at time k−1.
2. Propagate the particle sampled in step 1 using the process model.
3. Update the number of required particles. This is done by discretization of the state space into bins and counting the number of bins that include at least one particle. More bins containing particles means particles cover a larger part of the state space, hence, whenever the propagated particle from step 2 falls in an empty cell, the number of required particles is increased as described in [58].
  While resampling, the first particles will most likely fall into empty bins hence initially the number of required particles increases each time a particle is added. Later, more and more propagated particles fall into bins that already contain particles and the number of required particles will get updated less and less.
4. If the number of particles equals the number of required particles (or the maximum number of particles), stop sampling new particles.

For details, the reader is referred to [58]. A computationally attractive alternative for discretizing the entire state space in equally sized bins is the use of data structures that partition the state space more efficiently, e.g., KD trees [59]. In general, the number of particles will be increased in case the particles spread more and the number of particles will be descreased in case the particles are closer together. Inspired by [28,58] introduces an approach that is shown to be more efficient and simpler to implement.

1. Sample a particle index proportional to its weight using, e.g., multinomial resampling.
2. If the particle comes from a non-resampled bin, update the number of resampled bins, set the bin to being resampled and updated the required number of particles similar to [58].
3. If the number of particles equals the number of required particles (or the maximum number of particles), stop sampling new particles.

A modified version of the adaptive particle filter explained above can be found in [60]. A simpler approach is changing the number of particles in a way that ensures the sum of all particle likelihoods is fixed. This approach was taken in [61]. A survey that explains various less popular alternatives is given in [29,43] compares some methods that vary the number of particles on the fly. The works of [62,63] explain more recent variations of adaptive particle filters inspired by [28,58].

**Example** **11.** *The running example includes an adaptive particle filter as proposed by [58]. In order to investigate how the number of particles changes over time one simulation is done in which both the standard and adaptive particle filter get the same measurements and initial set of particles for estimating the robot pose over time.*

**Figure 9:**
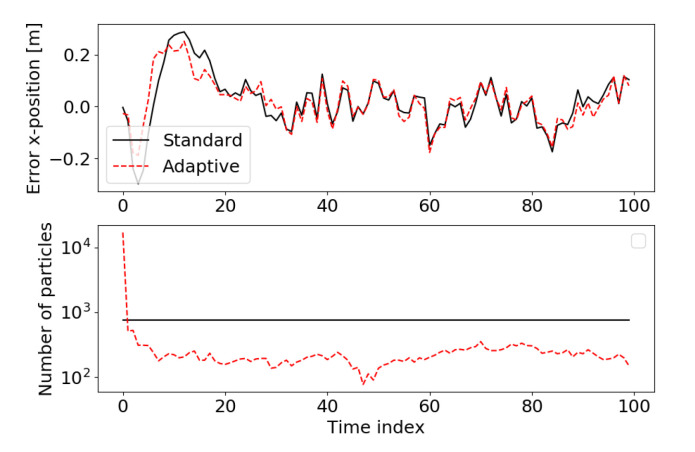
Example of how the adaptive particle filter achieves similar estimation accuracies (**top**) with a much lower number of particles (**bottom**). Black: standard particle filter, red: adaptive particle filter.

*Figure 9 shows the error in the x-position and the number of particles for both filters. The errors are roughly the same, however, the adaptive particle filter after initialization has approximately 100 to 300 particles, whereas the standard particle filter has a constant number of 750 particles. For reasons of completeness, the running example implements the adaptive particle filter proposed in [61] as well as the one proposed by [58] that was evaluated in Figure 9. Figure 9 is generated by running* *challenge5_adaptive_particle_filter.py*

#### 5.5.3. Other Ways to Manage Computational Costs

Many other ways exist to handle the computational costs of the particle filter. This section gives hints on further readings without going into details.

Depending on the type of particle filter, various parts of the computation can be done in parallel. Implementation on graphics processing units enables reducing computation time significantly, as is demonstrated in [24]. Parallel or distributed particle filters distribute particles among different processing units. How to distribute (route) particles and how much is gained for various routing policies is investigated in [64]. The work of [65] investigates using multiple local particle filters, that are correlated and low dimensional, in high dimensional problems using the phenomenon referred to as ’decay of correlations‘. The result is referred to as block particle filter.

Besides centralized resampling, where particle generation and weight computation can be done in parallel but resampling happens sequentially in a centralized computational unit, resampling algorithms and architectures for distributed particle filters are proposed in [66]. Here, the state space is partitioned into disjoint areas. The number of particles for each area is being computed and resampling happens in parallel in each of the areas.

For applications involving sensors networks, a central fusion center that runs an individual particle filter is not desirable for robustness, scalability and flexibility reasons [67]. For these problems, distributed particle filters are required, not only as a way to handle computational costs. Computing likelihoods while limiting communication in sensor networks requires likelihood consensus algorithms such as [68].

#### 5.5.4. Out-Of-Sequence Measurements (OOSMs)

In all of the above, the implicit assumption was that measurements $z_{1},z_{2},\ldots ,z_{k}$ arrive in the order in which they were recorded. In practice, however, this does not have to be the case.

In case the last particle filter update step has been performed at some time $t_{1}$, it is complicated to incorporate ‘older’ measurements recorded at time $t<t_{1}$. How to handle OOSMs depends on many problem specific characteristics, such as: how often are OOSMs expected? Is it acceptable to discard part of the measurement data? How much computation time and effort can be put in handling OOSMs? Did the last update step include resampling? Does the problem have characteristics that may help processing OOSM (more on that later)?

**Example** **12.** *Imagine the measurements in our running example are received from two different sensors on the robot: a 2D laser range finder and a camera. The data acquisition and processing of 2D laser data could be fast (e.g., few milliseconds) compared to the acquisition and processing of camera images that can be orders of magnitude larger in terms of data size (e.g., tens of milliseconds). As a result, a laser range finder measurement recorded a few milliseconds after a camera measurement may arrive at the particle filter algorithm before the camera-based measurement arrives. The result is an OOSM from the camera.*

The simplest way of handling OOSMs would be to check timestamps of incoming measurements and discard measurements older than the most recently processed measurement. This solution is acceptable in case OOSMs are rare and the effect of discarding OOSMs on the estimation accuracy is acceptable for the problem at hand. Due to its simplicity, this is the most popular way of handling OOSMs for practical problems.

The brute force approach would be storing historic states and measurements and redoing the estimation whenever an OOSM is received. This leads to increased and unpredictable computational costs and increased memory requirements. As a result, this solution is hardly ever used.

A pragmatic way to handle OOSMs could be to run the particle filter ‘in the past’:

1. Store all incoming measurements in a buffer, sorted on measurement acquisition time
2. Periodically check the measurement buffer, if a measurement is ϵ seconds old: process the measurement. Incoming measurements older than ϵ seconds are discarded.
3. The most recent particle filter posterior will be at least ϵ seconds old, use the process model to compute the estimate at the desired time whenever a more recent estimate is required.

The parameter ϵ can be defined based on typical sensor processing and acquisition times. This solution is simple and effective in case the process model is capable of delivering accurate predictions ϵ s ahead.

Whenever no resampling has happened, various alternative solutions are available for incorporating OOSMs. The approach of [69] proposes a way to deliver the optimal solution obtained from in-sequence processing without storing measurements. Main drawback is the increased computational complexity to $O(N_{s}^{3})$ or $O(N_{s}^{2})$, depending on the number of measurements received after the OOSM. In fact, real time execution of this solution is realistic for lower dimensional problems with a limited number of particles. Other, more heuristic based methods, like [70], can both lead to better or worse results then the simple approach described above.

Computational complexity of algorithms handling OOSMs can often be reduced at the expense of additional assumptions. The work of [71] offers a more efficient alternative to the optimal solution of [64] by assuming a Gaussian posterior. In [72], mixed linear/nonlinear state-space models are assumed such that Rao–Blackwellization can be used. Whether such assumptions are realistic depends on the specific problem at hand. In general, it is recommended to start with one of the simpler approaches to handle OOSMs and increase complexity of the solution whenever this is proven to be insufficient. The works of [69,72] and [71] and the references therein are recommended in that case.

## 6. Conclusions

The particle filter is among the most popular state estimation algorithms since its successful introduction in the early nineties [1]. Although the basics are well documented and available in many implementation examples, understanding and implementing the advancements made ever since is time consuming and non-trivial.

In an effort to present a single-entry point to those interested in applying particle filters to real-world problems while exploiting many of the advancements made in the last decades, this paper presented a hands-on tutorial. The code implementing the running example and many of the algorithms explained throughout this tutorial is publicly available [10]. This combination of properties distinguishes this work from other scientific works and open source repositories.

## Figures and Tables

**Table 1:** Simulation results for 100 simulations with 50 time steps each and three different resampling schemes. Errors are computed by taking the 2-norm of the error (true robot pose vs estimated robot pose). Presented are average error (mean), standard deviation (std. dev.) and the number of times a resampling step has been performed.

Scheme	Mean	Std. Dev.	# Resampling Steps
Every time step	3.73	2.97	5000
${\hat{N}}_{eff}$	3.81	2.98	1281
$1/\max_{i}\left(w_{k}^{i}\right)$	3.68	3.03	1806

**Table 2:** Average number of times each of the samples is selected in a test containing 100,000 trials (weights 0.366, 0.354, 0.119, 0.058, 0.102).

Resampling Alg.	Means				
Multinomial	1.83	1.77	0.60	0.29	0.51
Systematic	1.83	1.77	0.60	0.29	0.51
Stratified	1.83	1.77	0.60	0.29	0.51
Residual	1.83	1.77	0.59	0.29	0.50

**Table 3:** Standard deviation of the number of times each of the samples is selected in a test containing 100,000 trials (weights 0.366, 0.354, 0.119, 0.058, 0.102).

Resampling Alg.	Standard Deviations				
Multinomial	1.08	1.07	0.72	0.52	0.68
Systematic	0.77	0.76	0.69	0.51	0.65
Stratified	0.38	0.62	0.63	0.45	0.50
Residual	0.38	0.42	0.49	0.46	0.50

## Data Availability

Not applicable.
